# Die transformative Wirkung von künstlicher Intelligenz im Krankenhaus: Der Mensch steht im Mittelpunkt

**DOI:** 10.1007/s00108-023-01597-9

**Published:** 2023-10-18

**Authors:** Dominik Bures, Bernadette Hosters, Thomas Reibel, Florian Jovy-Klein, Johanna Schramm, Jennifer Brendt-Müller, Jil Sander, Anke Diehl

**Affiliations:** 1 Stabsstelle Digitale Transformation, Universitätsmedizin Essen, Hufelandstr. 55, 45147 Essen, Deutschland; 2 Stabsstelle Entwicklung und Forschung Pflege, Universitätsmedizin Essen, Essen, Deutschland; 3 Institut für Technologie- und Innovationsmanagement, Rheinisch-Westfälische Technische Hochschule Aachen, Aachen, Deutschland

**Keywords:** Digitalisierung, Diagnostik/künstliche Intelligenz, Künstliche Intelligenz/ethische Aspekte, Pflege, Smartes Krankenhaus, Digital transformation, Diagnostics/artificial intelligence, Artificial intelligence/ethics, Nursing, Smart hospital

## Abstract

Rasante Fortschritte der digitalen Technologie und die vielversprechenden Potenziale von künstlicher Intelligenz (KI) verändern unseren Alltag und haben längst im Krankenhaus Einzug gehalten. Gerade KI-Anwendungen bieten ein breites Spektrum an Einsatzmöglichkeiten und verfügen über ein beträchtliches Potenzial zur Verbesserung der medizinischen und pflegerischen Versorgung. In der radiologischen Diagnostik beispielsweise gibt es bereits vielfach gut erforschte Anwendungen zur KI-gestützten Bildauswertung. In dieser Arbeit werden weitere KI-Entwicklungen vorgestellt, die dazu beitragen können, das Gesundheitspersonal zu entlasten, um mehr Zeit für die direkte Patient*innenversorgung zu schaffen. Begleitend werden zentrale Aspekte rund um die Entwicklung und den Transfer von KI-basierten Anwendungen beleuchtet. Denn maßgeblich für die Integration von KI in die medizinische Praxis ist, dass sie mit äußerster Sorgfalt und Umsicht erfolgt. Datenschutz und ethische Aspekte dürfen keinesfalls vernachlässigt werden, und es ist von essenzieller Bedeutung, die Zuverlässigkeit und Integrität der KI-Systeme zu gewährleisten, um das Vertrauen sowohl der Patient*innen als auch des Gesundheitspersonals zu gewinnen. Eine umfassende Überprüfung auf mögliche Verzerrungen in den zugrunde liegenden Daten und Algorithmen ist dabei unverzichtbar. Im Spannungsfeld zwischen vielversprechenden Möglichkeiten und ethischen Herausforderungen kann die digitale Transformation in Medizin und Pflege zur Erhöhung der Patient*innensicherheit und zur Entlastung des Personals beitragen.

## Abstract (en)

Rapid advances in digital technology and the promising potential of artificial intelligence (AI) are changing our everyday lives and have already impacted on hospital procedures. The use of AI applications, in particular, enables a wide range of possible uses and has considerable potential for improving medical and nursing care. In radiological diagnostics, for example, there are already many well-researched applications for AI-based image evaluation. In this article further AI developments are presented, which can help to relieve medical staff in order to create more time for direct patient care. In addition, essential aspects regarding the development and transfer of AI-based applications are highlighted. It is crucial that the integration of AI into medical practice is carried out with the utmost care and prudence. Data protection and ethical aspects need to be considered and respected at all times. Ensuring the reliability and integrity of AI systems is essential to earn the trust of both patients and healthcare professionals. A comprehensive inspection for possible bias within the underlying data and algorithms is indispensable. In this field of tension between promising possibilities and ethical challenges, the digital transformation in medicine and care can be designed to increase patient safety and to relieve staff.

In der heutigen technologiegetriebenen Welt hat die Digitalisierung tiefgreifende Auswirkungen auf viele Sektoren, auch im Gesundheitsbereich. Künstliche Intelligenz (KI) ist dabei ein vielversprechendes Werkzeug mit dem Potenzial, diagnostische, therapeutische und pflegerische Prozesse zu verändern. An der Universitätsmedizin Essen (UME) werden KI-assoziierte Projekte auch bei Querschnittsaufgaben wie in der pflegerischen Versorgung oder Dokumentation getestet, um Potenziale zur Prozessverbesserung zu erforschen.

## Künstliche Intelligenz in der Krankenhaustransformation

**Figure 1:**
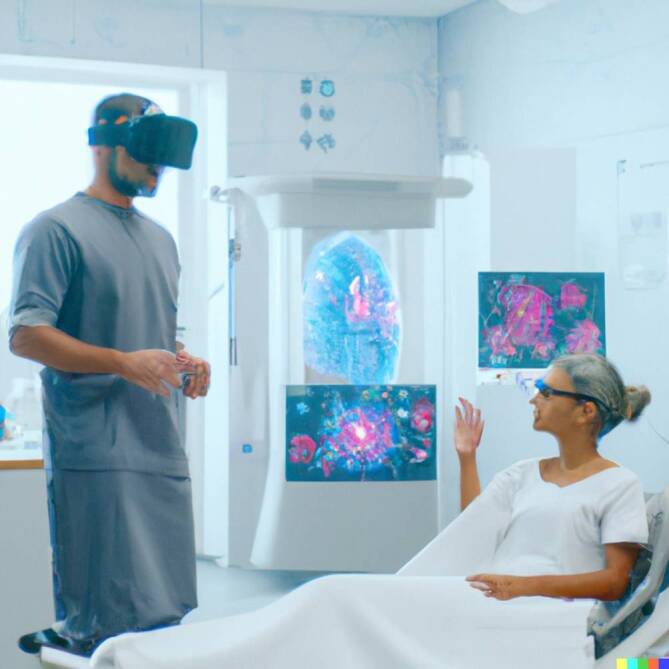

In der Radiologie ist die Nutzung von KI bereits etabliert und ermöglicht signifikante Verbesserungen in der Diagnostik [5]. Wie hoch die Wirkung von Digitalisierung und KI insgesamt sein wird, ist jedoch nicht absehbar. In Abb. 1 ist gezeigt, wie sich das von OpenAI entwickelte Programm DALL · E 2 beispielsweise den Blick in das Krankenhaus der Zukunft vorstellt.

Gesundheitspersonal muss nun auch zwischen Patient*innen und digitalen Systemen vermitteln

Ganz konkret eröffnet der Einsatz von KI-Anwendungen bei klinischen Prozessen Potenziale unter anderem in den Bereichen Fehlervermeidung, Effizienzsteigerung sowie Qualitätsverbesserung und kann dazu beitragen, die Zufriedenheit von Personal und Patient*innen zu erhöhen [25]. Darüber hinaus ermöglichen Dokumentationsunterstützung und digitale Assessment-Tools dem Fachpersonal eine verbesserte Einschätzung des individuellen Pflegebedarfs und die Auswahl geeigneter evidenzbasierter Pflegeinterventionen. Stetig verändern sich der Arbeitsalltag sowie die bisherigen Aufgabenbereiche: Medizinisches Personal muss nun auch zwischen Patient*innen und digitalen Systemen vermitteln [24].

Selbstverständlich spielen Digitalisierung und KI auch in der Krankenhausadministration, der Krankenhauslogistik sowie im Prozessmanagement eine zunehmende Rolle. Durch den Einsatz digitaler Technologien und KI-Anwendungen können effizientere und nachhaltigere Prozesse etabliert werden, die auch zu einer positiven Umweltbilanz führen [26].

## Der Weg zur Nutzung von künstlicher Intelligenz im Krankenhaus

Grundlage für die Entwicklung bzw. Nutzung von KI-Algorithmen in der Medizin ist die Verfügbarkeit der entsprechenden Datenbasis. Die sektorale Gliederung der Versorgung resultiert in aufgesplitterten, vielfach aufgrund von Interoperabilitäts- und Datenschutzhürden nicht kombinierbaren Gesundheitsdaten von Patient*innen. Dadurch fehlen insbesondere longitudinale Gesundheitsdaten, wie Verlaufsdaten der Laborparameter von Patient*innen.

Verfügbarkeit und Qualität von Daten sind essenziell für das effektive Training von KI-Algorithmen

Gleichzeitig sind Verfügbarkeit und Qualität von Daten essenziell, wenn KI-Algorithmen effektiv trainiert und aussagekräftige Ergebnisse erzielt werden sollen. Basis ist die Beachtung des FAIR-Prinzips bei Gesundheitsdaten. FAIR steht dabei für „findable, accessible, interoperable and reusable“ (auffindbar, zugänglich, interoperabel und wiederverwendbar) und legt Basisstandards für die Datenverfügbarkeit und -qualität fest [27].

**Figure 2:**
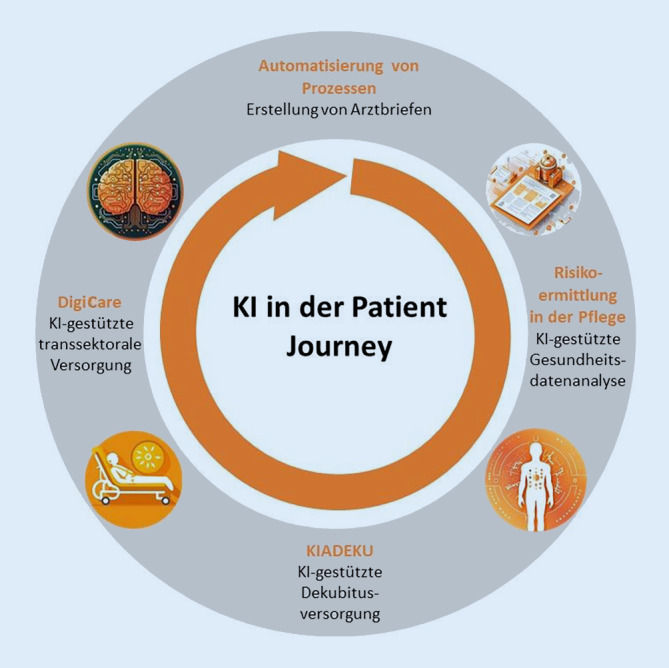

Die im Folgenden vorgestellten Projekte basieren auf Gesundheitsdaten, die im Rahmen der stationären Versorgung an der UME entstehen. Diese Daten sind interoperabel und stehen gemäß internationalen Standards über die UME-eigene Smart Hospital Information Platform (SHIP) nach streng geregelten, datenschutzkonformen Prozessen für die entsprechenden Projekte zur Verfügung. In Abb. 2 ist illustriert, an welchen Stellen die Projekte innerhalb einer exemplarischen „patient journey“ eingeordnet werden können.

## (Semi-)automatisierte Arztbrieferstellung

Bei der Versorgungsdokumentation entstehen viele Texte und Dokumente wie Befund- und Operationsberichte, Behandlungsdokumentationen sowie Entlassbriefe. Bei der Weitergabe von Informationen an andere Fachkolleg*innen werden jedoch nur Auszüge hiervon benötigt. Das Herausfiltern relevanter Informationen sowie das Schreiben von Texten sind zeitaufwendig, unter anderem da die Texte oft keiner einheitlichen Struktur folgen [15].

An dieser Stelle setzt ein Arbeitspaket des Konsortialprojekts SmartHospital.NRW an. Unter der Konsortialführung der UME arbeiten die Fraunhofer-Institute für Intelligente Analyse- und Informationssysteme (IAIS) und für Digitale Medizin (MEVIS), die Rheinisch-Westfälische Technische Hochschule (RWTH) Aachen, die Technische Universität (TU) Dortmund sowie die Unternehmen Dedalus und m.Doc an Transformationsmodellen für Krankenhäuser. So soll ein Tool für die KI-basierte semiautomatische Entlassbrieferstellung zur Entlastung des Personals entstehen. Zunächst werden Modelle aus dem Bereich des sogenannten „natural language processing“ trainiert, die in bestehenden Texten relevante Informationen und deren Zusammenhänge im Text erkennen können [1]. Im Vorfeld wurden dafür relevante Informationen, etwa aus einem Pathologiebefund, festgelegt. Das sind beispielsweise die Hauptdiagnose, bestimmte genomische Marker bzw. Antikörper sowie die zugehörigen Testergebnisse [2]. Um eine ausreichende Spezifität erreichen zu können, muss eine große Anzahl von Texten manuell gelabelt werden: Eine fachkundige Person markiert die relevanten Informationen und Zusammenhänge in Texten, um dem System das Erlernen dieser zu ermöglichen. Sobald ein Modell mit solchen Daten trainiert ist, kann es die relevanten Informationen und Zusammenhänge selbstständig als Vorschläge erkennen, sodass der Mensch lediglich eine Überprüfung durchführen muss. Die gefundenen Ergebnisse können dann in strukturierter Form, beispielsweise tabellarisch, wiedergegeben werden. Im zweiten Schritt können daraus neue Textabschnitte, wie eine Zusammenfassung, generiert werden. Auf diese Weise kann das KI-System beispielsweise Teile eines Entlassbriefs oder den gesamten Brief vorschreiben; der ärztliche Dienst muss den Text abschließend nur noch korrigieren und freigeben. Dadurch kann deutlich Zeit eingespart werden. Jedoch sind auch hier gut und umfassend trainierte Modelle essenziell, da Menschen das Schreiben von Fachtexten intuitiv oder basierend auf ihrem Fachwissen meistern, während es für Maschinen zum Teil noch eine Herausforderung darstellt.

## Künstliche Intelligenz in der Pflege

Obwohl die Profession der Pflege die größte Berufsgruppe im Gesundheitssektor bildet, konnte in einer systematischen Übersichtsarbeit aufgezeigt werden, dass sie bisher kaum aktiv an der Entwicklung, Umsetzung, Nutzung oder Bewertung von KI-Technologien beteiligt ist [21]. Dabei hat der Einsatz von KI grundsätzlich das Potenzial, eine individuelle Pflege zu ermöglichen, das Personal zu entlasten und die Versorgungsqualität zu verbessern [3, 25]. Die UME hat sich mit den nachfolgend vorgestellten Forschungsprojekten auf den Weg gemacht, das Potenzial von KI-Technologien für pflegerelevante Fragestellungen in der Point-of-care-Versorgung nutzbar zu machen.

### Auf künstliche Intelligenz gestützte Risikoermittlung in der Pflege

Neben Komplikationen durch Grunderkrankung, Operationen oder medizinische Therapien kann es während des stationären Aufenthalts zu weiteren unerwünschten Ereignissen („adverse events“ [AE]) kommen. Zu den häufigen AE gehört beispielsweise das Auftreten von Dekubitus, Deliren, Stürzen, Pneumonien oder Schmerzen. Um diesen vorzubeugen, werden im Rahmen des Pflegeprozesses Risikopatient*innen durch ein strukturiertes und standardisiertes Vorgehen erkannt und individuelle Pflegemaßnahmen abgeleitet [13]. Abgebildet wird dieser Prozess an der UME in einem durch elektronische Datenverarbeitung (EDV) gestützten Pflegeassessment, das täglich durchgeführt wird. Es besteht aus unterschiedlichen Items, die sich auf mehrere Dimensionen aufteilen und beispielsweise die Bewegungsfähigkeit oder den Ernährungszustand von Patient*innen abbilden. Dadurch ergeben sich unterschiedliche Triggerpunkte, die als Risikoindikatoren für die genannten AE dienen [13]. Trotz Evidenzbasierung bezieht sich das Pflegeassessment vorrangig auf Literaturarbeiten und schließt Interpretations- und Anwenderprobleme nicht aus [10].

In einem Arbeitspaket von SmartHospital.NRW wird auf Grundlage retrospektiver Daten die Zuverlässigkeit des beschriebenen Pflegeassessments hinsichtlich der Vorhersage pflegerelevanter Risiken validiert. Es wird unter Einbezug von soziodemografischen Merkmalen, Laborwerten, Vitalparametern und Medikation KI-gestützt analysiert, ob zusätzliche Muster zur AE-Prädiktion in den Daten gefunden werden können. Die Ergebnisse könnten prospektiv unter Hinzuziehung weiterer Daten, wie Herz- und Atemparameter, das Risikoassessment für AE verbessern.

### Personalisierte, evidenzbasierte Dekubitusversorgung

Die Anwendung von KI in der medizinischen Bildgebung ist weit verbreitet, beispielsweise in der Befundung von Röntgenbildern oder Mammographien [14, 17]. Obwohl ähnliche Potenziale in der pflegerischen Versorgung existieren und sich auf diesen Entwicklungen aufbauen ließe, wird die Technologie national noch nicht in der pflegerischen Versorgung eingesetzt. Das Verbundprojekt KIADEKU (KI-System zur Erkennung von Dekubitus und inkontinenzassoziierter Dermatitis), das von der UME geleitet wird und an dem die Ludwig-Maximilians-Universität (LMU) München und sciendis GmbH als Partner mitwirken, setzt hier an und rückt erstmalig pflegerische Fragestellungen zur Bildanalyse in den Fokus. Das Anwendungsfeld adressiert hierbei eine typische pflegerische Herausforderung – die Unterscheidung und Versorgung von Dekubitus und inkontinenzassoziierter Dermatitis (IAD; [16]). So sind sich die beiden Wunden zwar visuell ähnlich, benötigen aber eine unterschiedliche Behandlung, sodass Fehleinschätzungen zu Therapieverzögerungen führen.

Im Projekt KIADEKU wird ein KI-System zur digitalen Bildanalyse von Dekubitus und IAD entwickelt, um Pflegefachpersonen bei der Beurteilung und Dokumentation der Wundarten sowie bei personalisierten, evidenzbasierten Interventionen zu unterstützen. Hierfür wurde ein Minimaldatensatz erstellt und von Expert*innen evaluiert. Aktuell wird ein KI-Modell zur Klassifikation mit entsprechenden Bilddaten trainiert. Dieses soll in einen Demonstrator integriert und von Pflegefachpersonen am „point of care“ evaluiert werden. Das Projekt beinhaltet auch die Weiterentwicklung und Evaluation von Explainable-artificial-intelligence-Verfahren (erklärbare künstliche Intelligenz), um die Entscheidungen und Vorhersagen des KI-Modells transparent zu machen und die Akzeptanz beim Gesundheitspersonal zu verbessern.

### Die digitale „patient journey“ in der Onkologie

Symptombelastete Patient*innen, wie onkologische Patient*innen, sind häufig in ihrer Lebensqualität eingeschränkt, haben oft teils ungeplante stationäre Aufenthalte und leiden unter Therapieverzögerungen bzw. -abbrüchen, wodurch sich die Therapiekosten erhöhen [8, 19]. Eine adäquate Symptomkontrolle, insbesondere bei Palliativpatient*innen, bedarf einer dementsprechenden Versorgungsstruktur sowie des Selbstmanagements der Patient*innen über die Sektoren hinweg [4, 12, 18]. Eine interprofessionelle und sektorübergreifende Versorgungsstruktur, wie die Integration von „Advanced Practice Nurses“ (APN) und der Palliativmedizin, kommt in Deutschland allerdings nur selten zum Einsatz. Zudem fehlt es an entsprechenden informationstechnischen (IT) Infrastrukturen [12] zur transsektoralen Kommunikation in der Versorgung.

Im Verbundprojekt DigiCare entwickelt die UME mit der Universität Duisburg-Essen, der Hamburger Fern-Hochschule und m.Doc als Partnern eine E‑Health-Applikation und das Rollenprofil einer APN für ein transsektorales, interprofessionelles Symptommanagement. Die E‑Health-Applikation umfasst Funktionen wie Symptomerfassung, Chatbot, personalisierte Handlungsempfehlungen und ein Patient*innentagebuch. Dies wird teilweise durch KI unterstützt und wissenschaftlich evaluiert. Die UME bietet die passende IT-Infrastruktur mit digitaler Patient*innenakte, der Smart Hospital Information Platform (SHIP) und einer digitalen Symptomerfassung in der Onkologie. Die APN berät Patient*innen, vermittelt zwischen den Akteuren und begleitet die Pilot- und Feldstudie. Der interprofessionelle Ansatz ermöglicht eine evidenzbasierte, digitale Versorgungsstruktur.

Ein Ziel von DigiCare ist die Unterstützung der Patient*innen in ihrem Selbst- und Symptommanagement

Ziel ist die Unterstützung onkologischer Patient*innen in ihrem Selbst- und Symptommanagement sowie die Steigerung ihrer Lebensqualität. Die neue Versorgungsform wird so konzipiert, dass eine Übertragung auf weitere chronische Erkrankungen wie Herzinsuffizienz und auf weitere klinische Versorgungseinrichtungen möglich ist.

## Gestaltungsmöglichkeiten in der Versorgung

**Figure 3:**
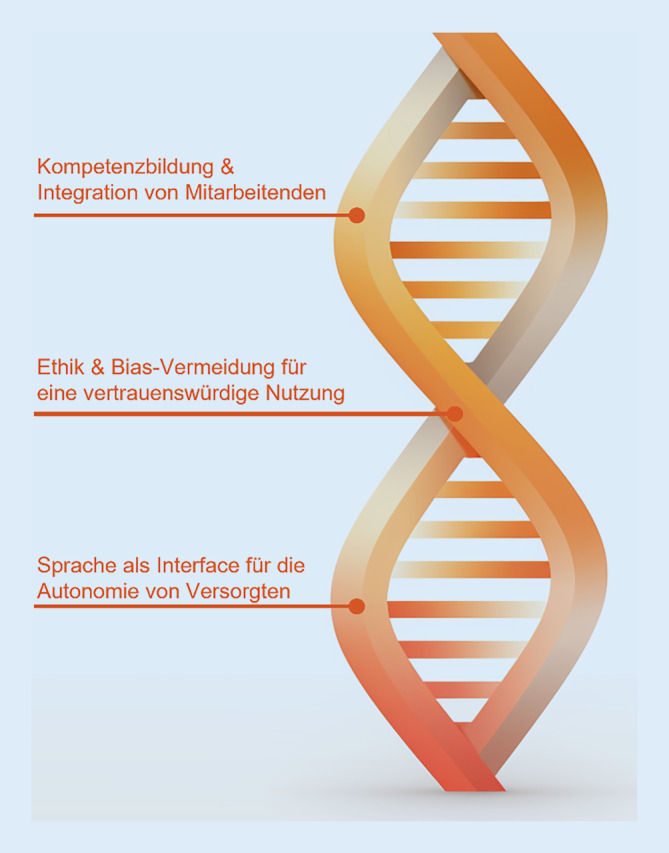

Die vorgestellten Projekte zeigen exemplarisch neue Einsatzmöglichkeiten von KI und Digitalisierung im Rahmen der „patient journey“ im Krankenhaus. Aktuelle Entwicklungen im Gesundheitswesen zielen darauf ab, die Voraussetzungen für einen umfassenden Einsatz von KI in Krankenhäusern zu schaffen [20]. Im Rahmen dreier Workshops, die mit klinischen Praktiker*innen und Akteur*innen (insgesamt 17 Teilnehmer*innen) durchgeführt wurden, konnten drei Schwerpunkte einer möglichen „patient journey“ der Zukunft identifiziert werden. In Abb. 3 findet sich eine Übersicht über diese drei Themenbereiche, die im Folgenden detailliert vorgestellt werden.

### Relevanz der Mitarbeiter*innenintegration

Es ist entscheidend, Mitarbeiter*innen in die Entwicklung und den Umgang mit KI einzubeziehen, um das Potenzial für die Patient*innenversorgung voll auszuschöpfen [23]. Die UME beispielsweise definiert neue Themenfelder und integriert Mitarbeiter*innen in das Change-Management, damit sie die digitale Transformation mitgestalten. Durch gezielte Implementierung von KI und Schulungen wird so den Mitarbeiter*innen ermöglicht, die Vorteile von KI zu nutzen und ihre Kompetenzen weiterzuentwickeln.

Neben den bekannten Leistungserbringern spielen Start-ups und etablierte Software‑, Medizintechnik- und Medizinprodukteanbieter [11] eine immer wichtigere Rolle in der Gestaltung der zukünftigen Krankenversorgung. Ihr Angebot an innovativen Technologielösungen und digitalen Services beinhaltet weitere Transformationsmöglichkeiten, wobei die Nutzung übergreifender Standards und die grundlegende Sicherstellung von Interoperabilität gewährleistet werden müssen.

### Sprache als Interface in der Versorgung

Die Implementierung von KI-gestützten Sprachassistenten im Krankenzimmer könnte Patient*innen in Zukunft mehr Autonomie in der eigenen Versorgung ermöglichen. In einem weiteren Arbeitspaket von SmartHospital.NRW wird eine KI-gestützte sprachgesteuerte Anwendung konzipiert. Diese soll Informationen zum Behandlungsprozess liefern und das Ausführen einfacher Tätigkeiten ermöglichen, beispielsweise die Bedienung der Sonnenblenden am Fenster. Sprachassistenten können das Gesundheitspersonal entlasten [6], indem sie sekundäre Aufgaben und administrative Tätigkeiten übernehmen. Dadurch können sich Mitarbeiter*innen idealerweise stärker auf die direkte Patient*innenversorgung und individuelle Betreuung der Patient*innen konzentrieren.

### Ethik und Bias-Vermeidung für empathische Zukunftsmedizin

Der Einsatz von KI in der Medizin erfordert die Berücksichtigung ethischer Fragen und möglicher Verzerrungen. Eine Prüfung auf „representation bias“, „measurement bias“, „aggregation bias“ oder „evaluation bias“ der zugrunde liegenden Daten ist unbedingt durchzuführen [7]. Generell ist eine regelmäßige Dokumentation, Überprüfung und Optimierung von Algorithmen und Modellen notwendig, um die Validität sicherzustellen. Daher ist eine Zusammenarbeit zwischen Fachkräften mit informationstechnischem und Fachkräften mit medizinischem Domänenwissen unausweichlich. Dies wird auch vom Deutschen Ethikrat [9] im Zusammenhang mit der Anwendung von KI-Systemen in der Medizin empfohlen.

Das Projekt ZERTIFIZIERTE KI beispielsweise entwickelt Zertifizierungsverfahren in Zusammenarbeit mit Partnern wie dem Bundesamt für Sicherheit in der Informationstechnik (BSI) und dem Deutschen Institut für Normung (DIN), um eine sichere und vertrauenswürdige Gestaltung von KI zu gewährleisten [22]. Workshops zu Ethik und Bias in der medizinischen KI-Anwendung wurden in diesem Rahmen in Kooperation mit der UME und dem Projekt SmartHospital.NRW erfolgreich durchgeführt. Ebenso spielt Datenschutzkonformität eine herausragende Rolle, um die Privatsphäre der Patient*innen zu schützen und den Missbrauch sensibler Daten zu verhindern. Nur die umfassende Berücksichtigung all dieser Prinzipien kann Vertrauen in die neuen Technologien schaffen, um eine erfolgreiche Weiterentwicklung und Akzeptanz von Digitalisierung und KI zu fördern.

## Fazit für die Praxis

- Anwendungen künstlicher Intelligenz (KI) im Krankenhaus sind vielseitig einsetzbar: neben der Bildgebungsanalyse etwa in der Dokumentation oder zur Unterstützung medizinischer und pflegerischer Entscheidungen.
- Die Zusammenarbeit von Gesundheits- und IT-Personal ist unerlässlich, um die Qualität der Nutzung zu gewährleisten. Sie minimiert potenzielle Fehler und verbessert die Versorgung.
- Die Implementierung von KI erfordert eine sorgfältige Planung, um Datenschutz und Zuverlässigkeit zu sichern sowie ethischen Herausforderungen zu begegnen. Eine Prüfung auf Bias ist unabdingbar.
- Krankenhäuser benötigen eine geeignete Infrastruktur für den reibungslosen Einsatz von KI. Die Nutzung standardisierter, valider, interoperabler Daten ist entscheidend für Effizienz und Genauigkeit der Systeme.
- Schulungen des Gesundheitsfachpersonals sind nötig, um eine effektive Nutzung und Weiterentwicklung von KI-Systemen sicherzustellen.
- Trotz Weiterentwicklung und Integration von KI im Krankenhaus steht der Mensch im Mittelpunkt.

## References

[1] Antweiler D, Beckh K, Chakraborty N (2023). Natural Language Processing in der Medizin.

[2] Antweiler D, Beckh K, Sander J (2020). Künstliche Intelligenz im Krankenhaus: Potenziale und Herausforderungen-Eine Fallstudie im Bereich der Notfallversorgung.

[3] Beck S, Faber M, Gerndt S (2023). Rechtliche Aspekte des Einsatzes von KI und Robotik in Medizin und Pflege. Ethik Med.

[4] Boland L, Bennett K, Connolly D (2018). Self-management interventions for cancer survivors: a systematic review. Support Care Cancer.

[5] Bonekamp D, Schlemmer H (2022). Artificial intelligence (AI) in radiology?: do we need as many radiologists in the future?. Urologe A.

[6] Cancio P, Morales G, Nhieu M (2023). Improving nurse and patient experiences with voice-controlled intelligent personal assistants. Nurse Lead.

[7] Cirillo D, Catuara-Solarz S, Morey C (2020). Sex and gender differences and biases in artificial intelligence for biomedicine and healthcare. NPJ Digit Med.

[8] Cleeland CS (2007). Symptom burden: multiple symptoms and their impact as patient-reported outcomes. J Natl Cancer Inst Monographs.

[9] Deutscher Ethikrat (2023). Mensch und Maschine – Herausforderungen durch Künstliche Intelligenz.

[10] Fiebig M, Hunstein D (2018). Digitale Dokumentation: Denkt künftig der Computer für mich?. Pflegez.

[11] Gehde KM, Rausch F, Leker J (2022). Business model configurations in digital healthcare—a German case study about digital transformation. Int J Innov Mgt.

[12] Girgis A (2020). The role of self-management in cancer survivorship care. Lancet Oncol.

[13] Hunstein D (2009). Das ergebnisorientierte PflegeAssessment AcuteCare (ePA-AC). Assessmentinstrument in der Pflege. Möglichkeiten und Grenzen.

[14] Kottner J, Kolbig N, Bültemann A (2020). Incontinence-associated dermatitis: a position paper. Hautarzt.

[15] Kreimeyer K, Foster M, Pandey A (2017). Natural language processing systems for capturing and standardizing unstructured clinical information: a systematic review. J Biomed Inform.

[16] Leblanc K, Alam T, Langemo D (2016). Clinical challenges of differentiating skin tears from pressure ulcers. EWMA J.

[17] Manava P, Galster M, Heinen H (2020). Algorithmen mit künstlicher Intelligenz: Entscheidungsunterstützung für Computertomographien des Thorax. Radiologe.

[18] Mccorkle R, Ercolano E, Lazenby M (2011). Self-management: enabling and empowering patients living with cancer as a chronic illness. CA A Cancer J Clinicians.

[19] Miaskowski C, Mastick J, Paul SM (2018). Impact of chemotherapy-induced neurotoxicities on adult cancer survivors’ symptom burden and quality of life. J Cancer Surviv.

[20] Nickel K, Milde K, Kremer D (2022). Bereit für das Smart Hospital? Whitepaper.

[21] O’Connor S, Yan Y, Thilo FJ (2022). Artificial intelligence in nursing and midwifery: a systematic review. J Clin Nurs.

[22] Poretschkin M, Schmitz A, Akila M (2021). Leitfaden zur Gestaltung vertrauenswürdiger Künstlicher Intelligenz (KI-Prüfkatalog).

[23] Ronquillo CE, Peltonen LM, Pruinelli L (2021). Artificial intelligence in nursing: priorities and opportunities from an international invitational think-tank of the nursing and artificial intelligence leadership collaborative. J Adv Nurs.

[24] Werner JA (2022). So krank ist das Krankenhaus: Ein Weg zu mehr Menschlichkeit, Qualität und Nachhaltigkeit in der Medizin.

[25] Werner JA, Forsting M, Kaatze T (2020). Smart Hospital: digitale und empathische Zukunftsmedizin.

[26] Werner JA, Kaatze T, Schmidt-Rumposch A (2022). Green Hospital: Nachhaltigkeit und Ressourcenschonung im Krankenhaus..

[27] Wilkinson MD, Dumontier M, Aalbersberg JI (2019). Addendum: the FAIR guiding principles for scientific data management and stewardship. Sci Data.

